# Association of *FOSL1* copy number alteration and triple negative breast tumors

**DOI:** 10.1590/1678-4685-GMB-2017-0267

**Published:** 2019-02-28

**Authors:** Leandro Tamião Rodrigues Serino, Tayana Schultz Jucoski, Stephanie Bath de Morais, Cíntia Callegari Coêlho Fernandes, Rubens Silveira de Lima, Cícero Andrade Urban, Luciane Regina Cavalli, Iglenir João Cavalli, Enilze Maria de Souza Fonseca Ribeiro

**Affiliations:** 1 Universidade Federal do Paraná Universidade Federal do Paraná Laboratory of Human Cytogenetics and Oncogenetics Departmentamento de Genética CuritibaPR Brazil Laboratory of Human Cytogenetics and Oncogenetics, Departmentamento de Genética, Universidade Federal do Paraná, Curitiba, PR, Brazil; 2 Hospital Nossa Senhora da Conceição Hospital Nossa Senhora da Conceição CuritibaPR Brazil Breast Unit, Hospital Nossa Senhora das Graças, Curitiba, PR, Brazil; 3 Georgetown University Georgetown University Department of Oncology WashingtonD.C. U.S.A. Department of Oncology, Lombardi Comprehensive Cancer Center, Georgetown University, Washington, D.C., U.S.A.

**Keywords:** Triple negative breast cancer, TNBC, DNA copy number alterations, CNA

## Abstract

Copy number alterations (CNAs) are a frequent feature in human breast cancer, and one of the hallmarks of genomic instability. The *FOSL1*, *GSTP1* and *CCND1* genes are located at 11q13, a cytoband commonly affected by CNA in breast cancer, with relevant function in progression and invasion. Our main goal was to analyze CNAs of these genes and determine their association with breast cancer subtypes. Seventy-three cases of invasive breast tumors [52 Luminal, 7 HER2+ and 14 triple negative (TNBC) subtypes] were analyzed by TaqMan assays. CNAs were observed for all genes, with gains more frequently observed. Gains of the *FOSL1* gene were observed in 71% of the cases. This gene was the only one with a statistically significant difference (*p*<0.001) among tumor subtypes, with increased copy number in TNBC compared to luminal and HER2+. No significant association of CNA and clinical and histopathological parameters from the patients was observed. Additional studies in larger breast cancer patient cohorts based on more refined molecular subtypes are necessary to confirm the observed association of *FOSL1* gain with aggressive breast tumors phenotypes.

## Introduction

Breast cancer is the cancer with the highest incidence among women worldwide. The incidence varies widely around the world, with rates from 19.3 new cases per 100,000 women in East Africa to 89.7 per 100,000 in Western Europe (Globocan, 2016). In Brazil, the estimate is of nearly 56 new cases per 100,000 women (INCA, 2016); about 50% of the cases and 58% of the deaths occur in low- and middle-income countries due to advanced stage diagnosis (WHO, 2016).

Based on gene expression arrays, breast cancers are classified into five major molecular subtypes: luminal A and B, basal, *HER2 (Human Epidermal Growth Factor Receptor 2)* positive and normal-like (Perou *et al.*, 2000; Sorlie *et al.*, 2003); other defined subtypes have also been identified in these studies including the interferon-rich, claudin low, and molecular apocrine (Farmer *et al.*, 2005; Prat *et al.*, 2010; Eroles *et al.*, 2012). These subtypes differ not only with regard to their pattern of gene expression and clinical features, but also in the response to treatment and clinical outcome (Sorlie *et al.*, 2003; Rouzier *et al.*, 2005; Sotiriou and Pusztai, 2009; Weigelt *et al.*, 2010; Martin *et al.*, 2011).

Although gene expression profiling has greatly contributed for the determination of breast cancer subtypes and its associated differential prognosis, at present, this defined “intrinsic” molecular classification is not routinely used in clinical practice to classify the patient’s breast tumor subtypes. Among the main reasons are the prohibitive costs of the equipment and reagents of the expression assays, and the lack of adequate technical personnel to conduct the complex informatics data analysis. A simplified clinicopathological classification, that defines subtypes based on the immunohistochemical analysis of ER, PR, HER2 receptors status and Ki-67 labeling index is instead adopted (Goldhirsch *et al.*, 2011, 2013). The breast cancer subtypes, Luminal A and B, HER2+, and triple negative breast cancer (TNBC), defined by this classification, are similar to the five main intrinsic subtypes, and represent a convenient approximation that can be performed in considerably less expensive and less complexes assays.

Copy number alterations (CNAs) are changes in gene copy number that have arisen in somatic tissue and are a frequent feature in human breast cancer, and one of the hallmarks of genomic instability (Hanahan and Weinberg, 2000, 2011). We recently investigated the copy number status of the genes *FOSL1*, *GSTP1* and *CCND1* in primary breast tumors with lymph node metastasis (Callegari *et al.*, 2016). These genes are mapped at 11q13 region, a cytoband commonly affected by CNA in breast cancer, and present relevant function in breast cancer progression and invasion (Santos *et al.*, 2008). The *FOSL1* gene belongs to the FOS family, which regulates several processes such as cell proliferation, differentiation, metastasis, angiogenesis, apoptosis and stimulating genes associated with hypoxia (Wisdom and Verma, 1993; Shaulian and Karin, 2002; Milde-Langosh, 2005; Kharman-Biz *et al.*, 2013). The Glutathione S-transferase pi 1 (*GSTP1*) gene belongs to the family of Pi class GSTs and is involved in cellular detoxification processes. High expression of GSTP1 was observed in cell lines treated with chemotherapy drugs, pointing to the potential involvement of this protein in tumor resistance to chemotherapeutic treatments (Batist *et al.*, 1986; Hayes and Pulford, 1995; Huang *et al.*, 2003). The *CCND1* gene encodes the Cyclin D1 protein, that plays an important role in cell cycle regulation, controlling the transition from the G1 to S phases (Massague, 2004). *CCND1* copy gain occurs in about 15 - 20% of breast tumors and its mRNA and protein was found overexpressed in approximately 50% of breast cancers, suggesting that other mechanisms than CNAs are involved in its expression regulation (Buckley *et al.*, 1993; Gillett *et al.*, 1994; Maia *et al.*, 2015).

In this study, we evaluated whether the CNAs present in these genes were associated with the IHC defined breast cancer major subtypes and with patients’ clinical and histopathological parameters.

## Material and Methods

### Sample Characterization

Seventy-three samples from primary breast carcinomas classified as invasive ductal carcinoma (IDC) were collected during primary surgery at the Hospital Nossa Senhora das Graças (HNSG), Curitiba, state of Paraná, South of Brazil, prior to any cancer treatment. The samples were collected with the patients’ written informed consent, and under approval of the local Ethical Committee in Human Research. The samples were immediately immersed in a transport medium, de-codified and sent without patients’ identifiers to the Laboratory of Human Cytogenetics and Oncogenetics (Genetics Department, Federal University of Parana).

Clinical and histopathological information regarding the age of patients, histological grade of tumors, and presence or absence of metastasis in axillaries lymph nodes, as well as immunohistochemical markers were retrieved from the pathological and medical reports (de-codified) and are summarized in Table 1. Samples were classified into subtypes Luminal A, Luminal B, HER2+, and triple negative breast cancer (TNBC) according to the status of hormone receptors ER and PR, Ki-67, and HER2 proteins, based on the St Gallen International Expert Consensus (Goldhirsch *et al.*, 2013). Based on ER and PR positivity (when ≥ 1% of tumor cells were immunoreactive according to Hammond *et al.* (2010), samples are classified in Luminal. The proliferation marker protein Ki-67 discriminate Luminal A (≤14%) and B (high), as well as the PR marker absent or low (indicating Luminal B with any Ki-67). Luminal B can also be divided in HER2 positive or negative (defined according to Wolff *et al.*, 2014). When positive, just the ER positivity is necessary to define Luminal B, with any Ki-67 and any PR result. Samples without expression of ER and PR are classified as HER2 positive (non-luminal) and TNBC (ER, PR and HER2 negative). In our sample, we noticed a great number of patients (see Suplementary Material Table S1) without the Ki-67 data, and even though these patients were classified individually, in the statistical analysis the group was described as “Luminal”, without the subdivision A e B. TNM classification was based on AJCC 8^th^ Edition (Amin *et al.*, 2016).

**Table 1:** Clinical and histopathological information.

	Sample size	Age (yrs)	Grade (%)	LN metastasis (%)	Lymphovascular invasion
ER^+^,PR^+^,HER2^-/+^	N=54	59 ± 14.55	Grade I (12.2)	Pos (52)	Present (47.8)
(Luminal)			Grade II (61.2)	Neg (48)	Absent (52.2)
			Grade III (26.5)		
ER^-^,PR^-^,HER2^+^	N=8	63 ± 14.66	Grade I (0)	Pos (87.5)	Present (16.7)
(HER2^+^)			Grade II (12.5)		
			Grade III (87.5)	Neg(12.5)	Absent (83.3)
ER^-^,PR^-^,HER2^-^	N=14	51 ± 10.17	Grade I (0)	Pos (42.8)	Present (64.3)
(TNBC)			Grade II (57.1)	Neg (57.2)	Absent (35.7)
			Grade III (42.8)		

ER: Estrogen receptor; PR: Progesterone receptor; HER2: Human Epidermal Growth Factor Receptor 2; LN: lymph node; Pos: lymph node positive; Neg: lymph node negative; TNBC: triple negative breast cancer.

### Copy number analysis

After confirmation of cancer diagnosis, the DNA was isolated by standard phenol-chloroform methods in snap frozen tissue samples. A pool of peripheral blood DNA from women with no cancer was used as control.

TaqMan® Copy Number Assays (Life Technologie) was used for the copy number analysis, using specific gene assays for *FOSL1*, *GSTP1* and *CCDN1*, as we previously described (Callegari *et al.*, 2016). *RNASE P* was used as a reference gene. The samples, including control DNA, were analyzed in triplicate using 96 well plates in the Viia 7 (Applied Biosystem) equipment. PCR conditions included an initial denaturation step at 95 °C for 10 min, followed by 40 cycles at 95 °C for 15 s and 60 °C for 1 min. Data analyses were performed using the Copy Caller software (Life Technologies). Samples that presented a CT value > 33 cycles and a z-score [#GTEQ#] 2.65 were not considered. The number of copies of the DNA of each sample (DNA test) was calculated in comparison with the control DNA; gains were considered for gene copy number of 2.51 or above, loss for 1.49 or below; and normal between 1.50 and 2.50.

### Statistical Analysis

The data were analyzed using the Kruskal-Wallis, Dunn, Student’s *t*, chi-square tests, and linear regression, with a statistical significance value set at *p*<0.05.

## Results

Seventy-three female patients, mean age 57.7 ± 13.7 (median 58 years old), diagnosed with primary breast cancer were studied. Clinico-pathological information is summarized in Table 1 and fully described in Table S1.

**Figure 1:**
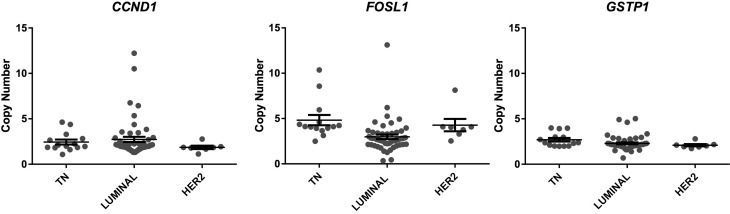
Copy number alterations (CNAs) of the *CCND1 FOSL1* and *GSTP1*genes according to the IHC defined breast tumor subtypes.

Copy number alterations (CNAs) were observed for all the genes analyzed in this study. Gains of copy number were the most frequent CNAs observed, most frequently for the *FOSL1* gene (71% of the cases), followed by *CCND1* (27% of the cases) and *GSTP1* (25% of the cases). Losses were observed in a lower frequency as follows: 6% for *FOSL1*, 3% for *GSTP1* and 10% for *CCND1*. For the *GSTP1* and *CCND1* genes there was no statistically significant difference among the CNAs observed and their distribution according to the IHC (Luminal, TNBC and HER2) defined tumor subtypes (χ^2^_2_=2.44; P>0,20 p=0.1130 and χ^2^_2_=0.43; P>0.80 *p*=0.3092 and χ^2^_2_=5.79; P>0.05, respectively) (Figure 1). For the *FOSL1* gene, the χ^2^ value is at the significance limit.

**Figure 2:**
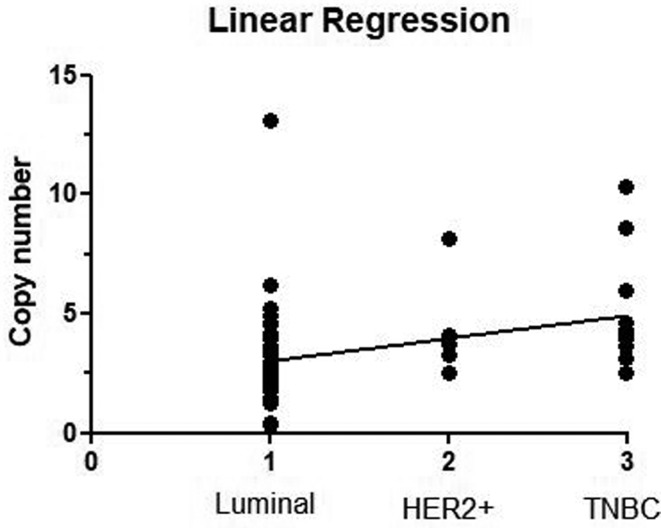
Distribution of the copy number alterations (CNAs) of the *FOSL1* gene and linear regression analysis according to the IHC defined breast cancer subtypes. Abbreviation: TNBC - *Triple Negative Breast Cancer*.

Related to *FOSL1* gene and IHC subtypes, CNAs were presented in an average of 2.98 ± 1.80 of the Luminal, 4.27 ± 1.79 of HER2, and 4.82 ± 2.14 of TNBC subtypes. These differences were tested by Fisher’s test. The Bartlett’s test showed that the variances were homogeneous (χ^2^_2 (corr)_=0.64; P>0.70), and the F-value was significant (F=6.08; P<0.05). Using Turkey’s test a significant difference was observed between the means of TNBC and Luminal subtypes (Δ=1.35<1.84). Linear regression test showed that CNAs in this gene were dependent of the tumor subtype (b=0.094 ± 0.27; *p*<0.001) (Figure 2).

We did not observe significant differences among the CNAs (normal vs. alterations) of the three genes analyzed and the patients’ clinical and histopathological parameters: mean age of patients under or over 50 years old (χ^2^_1_=0.11;P>0.70, χ^2^_1_=0.28; P>0.50 and χ^2^_1_=2.55; P>0.10), respectively for *FOSL1*, *GTSP1* and *CCND1*, tumor grade (I + II vs. III, (χ^2^_1_=0.98; P>0.30, χ^2^_1_=0.34; P>0.50 and χ^2^_1_=0.03; P>0.80, respectively), and lymph node metastasis status (χ^2^_1_=1.90; P>0.10, χ^2^_1_=0.58; P>0.30, and χ^2^_1_=1.05; P>0.30, respectively).

In addition, we did not observe significant differences among the three subtypes of tumors (Luminal, HER2 and TNBC) and lymphovascular invasion (χ^2^_*2*_*=*2.84; P>0.20) and presence of distant metastasis (χ^2^_*2*_=3.60; P>0.10), but a significant difference was observed for grade I+II vs. III (χ^2^_*2*_=8.51; P<0.05). The difference was mainly due to the value of the partial χ^2^ (6.87/8.51=81%) observed in the HER2 subtype and, probably, due to the low number of cases analyzed.

Finally, according to the data described in Table S1, 55 patients presented follow up information (41 classified as luminal, 4 HER2 and 10 TNBC). The mean of the clinical follow up time of the 41 luminal patients was 86.24 ± 45.32 months, of the 4 HER2 patients was 41.25 ± 33.17 months and of the 10 TNBC patients was 62.7 ± 38.6 months. In each subtype, two patients died, respectively 5%, 50%, and 20% in the luminal, HER2, and TNBC subtypes. As expected, the number of patient deaths was not equally distributed in the three subtypes of tumors (χ^2^_*2*_= 8.6; P<0.05), but the difference was mainly due to the value of the partial χ^2^ (6.22 /8.60=72%) observed in the HER2 subtype that presented a low number of the patients. The difference between the means of the clinical follow up time observed in the subtypes luminal and TNBC was not significant (*t*=1.51; P>0.10). Thirty-seven patients (57%) were alive and with no evidence of disease (NED) and 18 (33%) presented some event (EV), like death, local relapse, or distant metastasis (Table S1). We did not observe a differential distribution of CNAs among the patients in the two groups (54 and 26 CNAs respectively for NED and EV), but we could notice that the *FOSL1* gene showed the highest frequency of CNAs (26 and 17 respectively for NED and EV), with predominance in the two more aggressive subtypes, HER2 and TNBC (Table S1).

## Discussion

In this study, we evaluated the copy number of *FOSL1*, *GSTP1, CCND1* in different subtypes of breast carcinomas, classified according to the status of hormone receptors, ER and PR, KI-67, and HER2 protein. It is important to address that the classification based on immunohistochemistry (IHC) is similar but not identical to intrinsic subtypes (using genetic array testing) and represent a convenient approximation. For example, the IHC subtype TNBC overlaps 80% with the intrinsic “basal-like” subtype, but includes some special histological types such as medullary and adenoid cystic carcinoma (Goldhirsch *et al.*, 2011).

The genes were selected based on their critical roles in breast cancer, as well as on our previous study on primary breast cancer, showing the preferentially involvement of the 11q13 region, where these genes are mapped, in copy number alterations (CNAs) (Santos *et al.*, 2008).

Here were observed CNAs for all the three genes evaluated, with gains being the predominant change (71% for *FOSL1*, 27% for *CCND1,* and 25% for *GSTP1*). Considering the physical proximity of these genes in 11q13, they could simultaneously be affected by copy number gain/amplification or loss/deletion. In our samples, we observed that gains, losses, or no alterations of these genes were not equally distributed (χ^2^ = 40.05, P<0.001) mainly due to the *FOSL1* result, but corroborating this hypotheses.

The *FOSL1* gene was observed with the highest frequency of CNAs in our study and the only one with a significant difference among subtypes, more specifically between TNBC and Luminal subtypes. In addition, among patients with follow up, *FOSL1* showed the highest CNAs frequency in the two more aggressive subtypes, HER2 and TNBC. To our knowledge although there are several reports on *FOSL1* gene expression changes, there are no reports in relation to its copy number. Since the report of Kustikova *et al.* (1998), the correlation of *FOSL1* expression and mesenchymal characteristics of epithelial tumors is well accepted. Overexpression in epithelioid carcinoma cells greatly influences cell morphology, motility, and invasiveness. Belguise *et al.* (2005) induced the overexpression of *FOSL1* in the MCF-7 cell line (ER+ and less aggressive) and the subexpression in the MDA-MB-231 (TN phenotype and more aggressive) showing that the modulation of *FOSL1* expression directly affected cell proliferation, invasiveness, and motility of these cells *in vitro*. In the same direction, Kharman-Biz *et al.* (2013) and Zhao *et al.* (2014) independently observed that *FOSL1* expression was higher in TNBC compared to luminal tumors. These and other studies indicate overall that tumor cells with high metastatic capabilities present higher expression of *FOSL1* (Zajchowski *et al.*, 2001; Zhao *et al.*, 2014). Although we did not perform an expression analysis of this gene, based on our findings we suggest that CNAs of *FOSL1* can be one of the mechanisms that lead to the reported overexpression of this gene in aggressive breast tumors, such as TNBC. Data from our research group (Callegari *et al.*, 2016), however, did not find a specific correlation between *FOSL1* copy number and mRNA expression in breast tumors in general, although only a small number of samples was analyzed (n=31).

The *GSTP1* gene was observed with normal copies in 72% of the breast tumor cases evaluated in this study and gain in 23% of the cases. No significant difference for this gene was observed in relation to its copy number and tumor subtypes and/or clinical-pathological parameters. The described alteration of the GSTP1 enzyme in breast tumors can be due to the presence of polymorphisms in this gene, such as the Ile105Val polymorphism, where the homozygous Ile has been shown to confer increase in its enzymatic activity (Ünlü *et al.*, 2008; Khabaz, 2014). Data from our group described in Torresan *et al.* (2008) studying the same polymorphism in Euro-descendant patients in southern Brazil, found a positive association between the Val allele and the risk of breast cancer when combined with polymorphisms in the CYP genes. However, others found no association between the Ile105Val polymorphism and breast cancer risk (Ünlü *et al.*, 2008; Khabaz, 2014).

The *CCND1* gene was observed with a copy number gain in 26% of our cases. This data is consistent with other studies that found amplification of this gene in approximately 15-20% of breast tumors (Gillett *et al.*, 1994; Holm *et al.*, 2012; Burandt *et al.*, 2014). However, we did not find any association of CNAs in this gene with the breast cancer subtypes and clinical and histopathological parameters from the patients.

In conclusion, we showed in this study that the *FOSL1, GSTP1,* and *CCND1* genes present gains of copy number in invasive breast tumors. Regression analysis showed that CNAs of the *FOSL1* gene were significantly dependent of the tumor subtype TNBC when compared to the luminal tumors, suggesting its association with aggressive breast tumor phenotypes. Additional studies in larger breast cancer patient cohorts and classified based on the more refined molecular subtypes, are necessary to confirm these findings.

## Supplementary material

The following online material is available for this article:

Table S1 Clinico-pathological information, classification, follow up and DNA copy number of 73 patients.
