# Supplementary material for: Association of FOSL1 copy number alteration and triple negative breast tumors
Source: Genet Mol Biol. 2019 Feb 28;42(1):26–31. doi: 10.1590/1678-4685-GMB-2017-0267 (PMC6428133; doi:10.1590/1678-4685-GMB-2017-0267)
Supplement: Supplementary file 1 [file 1415-4757-GMB-1678-4685-GMB-2017-0267-20190129-suppl1.pdf]

Genetics and Molecular Biology

Supplementary Materials to: "Association of *FOSL1* copy number alteration and triple negative breast tumors"

Table S1 - Clinico-pathological information, classification, follow up and DNA copy number of 73 patients.

Sample	Age (yrs)	Grade	TNM*	Lympho vascular invasion	ER	PR	ERBB2/ HER2	Ki67 (%)	IHC classification**	Follow up (mo)	Event	CN GSTP1	CN CCND1	CN FOSL1
**1**	59	II	T2N1aMx	POS	NEG	NEG	NEG	NA	TNBC	159	NED	N	L	G
**2**	42	III	T2N1aMx	POS	NEG	NEG	NEG	NA	TNBC	73	Death Met lung and CNS	N	N	G
**3**	45	II	T2N1b2Mx	POS	NEG	NEG	NEG	NA	TNBC	NA	NA	G	N	G
**4**	51	II	T2N0Mx	NEG	NEG	NEG	NEG	NA	TNBC	43	NED	G	G	G
**5**	51	II	NA	NEG	NEG	NEG	NEG	NA	TNBC	62	Met liver	N	N	N
**6**	43	II	T2N0Mx	POS	NEG	NEG	NEG	NA	TNBC	64	NED	G	N	G
**7**	43	III	T3N1bIMx	POS	NEG	NEG	NEG	NA	TNBC	NA	NA	G	G	G
**8**	59	II	T2N1Mx	POS	NEG	NEG	NEG	NA	TNBC	NA	NA	N	N	G
**9**	66	II	T2N0Mx	POS	NEG	NEG	NEG	NA	TNBC	NA	NA	N	N	G
**10**	62	II	T1N0M0	NEG	NEG	NEG	NEG	10-15	TNBC	59	Local Relapse	N	N	G
**11**	66	III	NA	POS	NEG	NEG	NEG	80	TNBC	73	Death	N	G	G
**12**	58	III	T2N0M0	NEG	NEG	NEG	NEG	42	TNBC	43	NED	G	G	G
**13**	35	III	NA	NEG	NEG	NEG	NEG	80	TNBC	12	Met CNS	N	N	G
**14**	41	III	NA	POS	NEG	NEG	NEG	50	TNBC	39	NED	G	G	G
**15**	44	III	T3NIbIVMx	POS	POS	POS	NEG	NA	LA OR B	127	NED	N	L	G
**16**	54	II	T2N2Mx	POS	POS	POS	NEG	NA	LA OR B	17	Met bone and lung	N	L	G
**17**	34	NA	T4bN1bIIIMx	POS	POS	NEG	NEG	NA	LB	107	Met bone	N	N	N
**18**	38	II	T2N1Mx	POS	POS	POS	NEG	NA	LA OR B	96	Met bone	N	N	G
**19**	89	III	T2N2aMx	POS	POS	POS	NEG	NA	LA OR B	84	NED	G	N	G
**20**			T1N0Mx	NEG	POS	POS	NEG	NA	LA OR B	NA	NA	N	N	N
**21**	76	I	T1N0Mx	NEG	POS	POS	NEG	NA	LA OR B	108	NED	N	N	N
**22**	62	III	NA	POS	POS	POS	NEG	NA	LA OR B	37	Met bone and liver	N	N	N
**23**	33	III	T2N0Mx	NEG	POS	POS	NEG	NA	LA OR B	73	NED	N	N	G
**24**	67	III	T4bN1aMx	POS	PO	POS	NEG	NA	LA OR B	60	NED	N	N	G
**25**	44	II	T1cN1M0	NEG	POS	POS	NEG	20-30	LB	92	Local relapse, lung met?	N	N	G
**26**	62	II	T3N2Mx	POS	POS	POS	NEG	NA	LA OR B	NA	NA	N	N	G

Sample	Age (yrs)	Grade	TNM*	Lympho vascular invasion	ER	PR	ERBB2/HER2	Ki67 (%)	IHC classification**	Follow up (mo)	Event	CN GSTP1	CN CCND1	CN FOSL1
**27**	72	II	T2N0Mx	NEG	POS	POS	NEG	5	LA	NA	NA	N	N	N
**28**	86	III	NA	NA	POS	POS	NEG	20	LB	65	NED	G	G	G
**29**	73	II	NA	POS	POS	POS	NEG	5-10	LA	90	NED	N	G	N
**30**	46	III	T2N1Mx	POS	POS	POS	NEG	50	LB	92	NED	N	N	N
**31**	81	II	NA	NA	POS	POS	NEG	10	LA	2	NED	N	N	N
**32**	58		NA	NA	POS	POS	NEG	20	LB	63	NED	G	G	G
**33**	42	II	NA	NEG	POS	POS	NEG	20	LB	82	NED	N	N	G
**34**	70	II	NA	POS	POS	POS	NEG	30	LB	32	NED	G	G	G
**35**	78	III	T1N0M0	POS	POS	POS	NEG	10	LA	59	NED	N	G	N
**36**	57	I	T1cN0M0	NEG	POS	POS	NEG	25	LB	46	NED	G	G	G
**37**	48	II	NA	NEG	POS	POS	NEG	60	LB	43	NED	G	G	G
**38**	59	II	NA	NEG	POS	POS	NEG	5-10	LA	167	Local relapse	N	L	G
**39**	68	II	NA	NEG	POS	POS	NEG	14	LA	35	NED	N	L	N
**40**	52	I	NA	NEG	POS	POS	NEG	18	LB	39	NED	G	G	N
**41**	79	I	T1cN0Mx	NEG	POS	POS	POS	NA	LB	187	NED	N	N	L
**42**	53	III	T2N1M0	POS	POS	POS	POS	NA	LB	187	NED	N	N	L
**43**	50	III	T1cN0M0	NEG	POS	POS	POS	NA	LB	120	NED	N	N	N
**44**	71	II	T4N3	POS	POS	POS	POS	NA	LB	55	Generalized met	L	G	L
**45**	41	II	T2N3	NEG	POS	POS	POS	NA	LB	73	Death	N	N	G
**46**	72	I	T2N0M0	NEG	POS	POS	POS	NA	LB	122	Met liver	L	L	L
**47**	63	II	T2N0M0	NEG	POS	POS	NEG	NA	LA OR B	139	NED	G	G	G
**48**	56	II	T2N1M0	POS	POS	POS	POS	NA	LB	142	NED	N	N	G
**49**	51	III	NA	NEG	POS	POS	POS	NA	LB	110	NED	N	N	G
**50**	47	III	T2N1M0	NA	POS	NEG	POS	NA	LB	151	NED	N	N	G
**51**	54	II	T2N0Mx	POS	POS	POS	POS	NA	LB	NA	NA	N	N	G
**52**	46	III	T1cN0M0	NEG	POS	POS	POS	NA	LB	119	NED	N	G	G
**53**	59	II	T2N0M0	NEG	POS	POS	POS	NA	LB	115	NED	N	N	N
**54**	44	II	T4N0Mx	NEG	POS	POS	POS	NA	LB	NA	NA	N	N	G
**55**	82	II	T4bN0Mx	NA	POS	POS	POS	NA	LB	NA	NA	N	N	G
**56**	60	II	T1cN1M0	POS	POS	POS	NEG	NA	LA OR B	116	NED	N	N	G
**57**	61	II	NA	POS	POS	POS	POS	NA	LB	55	Death	N	N	G
**58**	44	II	NA	NEG	POS	POS	POS	NA	LB	5	NED	N	N	N
**59**	70	NA	T2N3aMx	POS	POS	POS	POS	NA	LB	NA	NA	N	N	G
**60**	42	III	TisNxMx	POS	POS	POS	POS	NA	LB	NA	NA	N	N	G
**61**	72	II	T2N2Mx	POS	POS	POS	NA	NA	LA OR B	60	NED	G	G	G

Sample	Age (yrs)	Grade	TNM*	Lympho vascular invasion	ER	PR	ERBB2/HER2	Ki67 (%)	IHC classification**	Follow up (mo)	Event	CN GSTP1	CN CCND1	CN FOSL1
**62**	66	I	T2NxMx	POS	POS	POS	NEG	5-8	LA	98	NED	G	G	G
**63**	57	II	T2N0M0	NEG	POS	POS	POS	5-8	LB	96	Contralateral breast cancer	G	G	G
**64**	62	I	T1cN0Mx	POS	POS	POS	POS	NA	LB	NA	NA	N	N	G
**65**	45	II	NA	POS	POS	POS	NEG	10	LA	56	NED	N	N	N
**66**	42	II	NA	NA	POS	POS	POS	5	LB	NA	NA	N	N	N
**67**	63	III	T2N1bIII	POS	NEG	NEG	POS	NA	HER2	84	Death ovarian cancer	N	N	G
**68**	72	III	T4N3	POS	NEG	NEG	POS	NA	HER2	15	Death	N	N	G
**69**	55	III	T1cN1	POS	NEG	NEG	POS	NA	HER2	51	Met liver	N	L	G
**70**	79	III	T2N0Mx	NEG	NEG	NEG	POS	NA	HER2	15	NED	N	N	G
**71**	58	II	T3N1aMx		NEG	NEG	POS	NA	HER2	NA	NA	N	N	G
**72**	89	III	T4/R2NxM0	POS	NEG	NEG	POS	NA	HER2	NA	NA	G	G	G
**73**	84	III	T2N2Mx	POS	NEG	NEG	POS	60	HER2	NA	NA	N	N	G

NA, not available; POS, positive; NEG, negative; ER, estrogen receptor; PR, progesterone receptor; ERBB2/HER2, overexpression of HER2 protein; LA, luminal A; LB, luminal B; HER2, HER2 positive; TNBC, triple negative breast cancer; NED, no evidence of disease; met, metastasis; CN, copy number; G, gain; L, loss; N, normal.

*Based on AJCC Cancer Staging Manual, Eighth Edition.

** Based on Goldhirsch *et al*., 2013.
